# Effect of ramp slope on intensity thresholds based on correlation properties of heart rate variability during cycling

**DOI:** 10.14814/phy2.15782

**Published:** 2023-08-07

**Authors:** Pablo R. Fleitas‐Paniagua, Rafael de Almeida Azevedo, Mackenzie Trpcic, Juan M. Murias, Bruce Rogers

**Affiliations:** ^1^ Faculty of Kinesiology University of Calgary Calgary Canada; ^2^ Applied Physiology and Nutrition Research Group ‐ Center of Lifestyle Medicine, Faculdade de Medicina FMUSP Universidade de São Paulo São Paulo Brazil; ^3^ College of Health and Life Sciences Hamad Bin Khalifa University Doha Qatar; ^4^ College of Medicine University of Central Florida Orlando Florida USA

**Keywords:** cycling ramp, endurance exercise, exercise thresholds, heart rate variability, intensity distribution

## Abstract

An index of heart rate variability (HRV), detrended fluctuation analysis (DFA a1) has gathered interest as a surrogate marker of exercise intensity boundaries. The aim of this report was to examine heart rate variability threshold (HRVT) behavior across different ramp incremental (RI) slopes. Seventeen participants completed a series of three RI (15, 30, and 45 W · min^−1^ slopes) with monitoring of gas exchange parameters, heart rate (HR) and HRV. HRVT1 was defined as the V̇O_2_ or HR at which DFA a1 reached 0.75 and the HRVT2 at which these values reached 0.5. HRVTs were compared by Pearson's *r*, Bland–Altman analysis, ICC_3,1_, ANOVA, and paired *t*‐testing. An excellent degree of reliability was seen across all three ramps, with an ICC_3,1_ of 0.93 and 0.88 for the HRVT1 V̇O_2_ and HR, respectively, and 0.90 and 0.92 for the HRVT2 V̇O_2_ and HR, respectively. Correlations between HRVT1/2 of the individual ramps were high with *r* values 0.84–0.95 for both HR and V̇O_2_. Bland–Altman differences ranged between −1.4 and 1.2 mL · kg^−1^ · min^−1^ and −2 and +2 bpm. Paired *t*‐testing showed no mean differences between any HRVT1/2 ramp comparisons. Cycling ramp slope does not appear to affect either HRVT1 or HRVT2 in terms of HR or V̇O_2_.

## 1 INTRODUCTION

As intensity rises during incremental exercise testing, measurements of cardiopulmonary factors such as heart rate (HR), ventilation, oxygen uptake (V̇O_2_), and carbon dioxide (V̇CO_2_) production, all change in patterns that allow for useful physiologic assessment (Beltz et al., 2016; Black et al., 2017; Iannetta et al., 2020, 2022; Jamnick et al., 2020; Jones et al., 2019; Keir et al., 2022; Pettitt et al., 2013; Poole & Jones, 2012). These sets of relationships have formed a conceptual framework for the purpose of determining exercise threshold locations. Two general boundary areas have been recognized, separating exercise intensity into three domains: moderate, heavy, and severe (Poole & Jones, 2012). The first boundary, separating the moderate from the heavy intensity domain, represents the divergence in exercise‐related V̇CO_2_ production relative to V̇O_2_ uptake, which results in an increase in ventilation in relation to V̇O_2_ and which also coincides with the beginning of blood lactate elevation above baseline. This has been referred to as the gas exchange threshold (GET), the first ventilatory (VT1), or lactate threshold (LT1), respectively (Beltz et al., 2016; Keir et al., 2022; Pettitt et al., 2013; Poole & Jones, 2012). The second intensity boundary, separating the heavy from the severe intensity domain, represents the maximal metabolic steady‐state which is sometimes referred to as the “critical” intensity where cardiopulmonary and metabolic homeostasis is no longer possible (Iannetta et al., 2018, 2020, 2022; Jones et al., 2019; Poole & Jones, 2012). Although the concepts of critical power and maximal lactate steady state are often considered as the best estimators of this boundary (Iannetta et al., 2022), they are quite demanding from a time and effort perspective. During incremental testing, the metabolic rate at which this boundary occurs can be derived from the second ventilatory (VT2), lactate threshold (LT2), or the respiratory compensation point (RCP) (Beltz et al., 2016; Jamnick et al., 2020; Jones et al., 2019; Keir et al., 2022; Pettitt et al., 2013; Poole & Jones, 2012). Exercise ramp protocols used to determine these metrics can be performed with different rates of intensity increase described through a measure of “slope” (Beltz et al., 2016; Iannetta et al., 2020; Poole & Jones, 2012). For example, a given ramp may have a steep “slope” where the intensity rises rapidly or conversely, as having a shallow slope where the intensity rises slowly. Prior research has shown that certain measured parameters are usually independent of ramp slope, including the maximal V̇O_2_ attained (V̇O_2MAX_), as well as both VT1/GET and VT2/RCP if measured as the corresponding HR or V̇O_2_ (Davis et al., 1982; Weston et al., 2002). However, this is not necessarily the case if thresholds are measured by external load markers such as cycling power (Boone & Bourgois, 2012; Iannetta et al., 2019; Keir et al., 2018).

Over the past two decades, the use of heart rate variability (HRV) has gathered interest as a surrogate method in determining thresholds which demarcate exercise intensity boundaries (Cottin et al., 2006; Gronwald et al., 2020; Karapetian et al., 2008; Mateo‐March et al., 2022; Michael et al., 2017; Naranjo‐Orellana et al., 2021; Rogers et al., 2021a; Rogers, Giles, et al., 2021; Rogers & Gronwald, 2022; Rogers, Mourot, & Gronwald, 2021; Schaffarczyk et al., 2023). HRV in general refers to the various statistical patterns in the cardiac beat‐to‐beat time sequence. As exercise intensity rises, there is a reciprocal change in autonomic nervous system (ANS) balance consisting of parasympathetic withdrawal and sympathetic enhancement (White & Raven, 2014). This in turn leads to effects on the cardiac pacemaker cells through the vagal system with resultant variation in cardiac beat‐to‐beat timing and HR elevation. As opposed to cardiopulmonary (V̇O_2_) or metabolic (lactate) parameters, HRV represents shifts in ANS balance that can be seen during both rest and exercise. Multiple studies have shown utility of various HRV parameters including linear, frequency‐related, and nonlinear indexes to aid in the identification of both the VT1/LT1 and VT2/LT2 (Cottin et al., 2006; Gronwald et al., 2020; Karapetian et al., 2008; Mateo‐March et al., 2022; Michael et al., 2017; Naranjo‐Orellana et al., 2021; Rogers et al., 2021a; Rogers, Giles, et al., 2021; Rogers & Gronwald, 2022; Rogers, Mourot, & Gronwald, 2021; Schaffarczyk et al., 2023). However, most HRV indexes reach a nadir value at the VT1/LT1 making them sub optimal for comprehensive threshold investigation (Cottin et al., 2006; Karapetian et al., 2008). However, a nonlinear index based on the short‐term scaling exponent of detrended fluctuation analysis (DFA a1) has shown potential as a marker for exercise intensity encompassing both threshold boundaries (Gronwald et al., 2020; Mateo‐March et al., 2022; Naranjo‐Orellana et al., 2021; Rogers et al., 2021a; Rogers, Giles, et al., 2021; Rogers & Gronwald, 2022; Rogers, Mourot, & Gronwald, 2021; Schaffarczyk et al., 2023). DFA a1 reflects the degree of fractal organization and correlation of the cardiac beat‐to‐beat pattern over various times scales (Hardstone et al., 2012). At low‐exercise intensity, values are typically well correlated (DFA a1 values at or above 1.0), then decrease through the moderately correlated range near the VT1/LT1 (about 0.75), become uncorrelated close to the VT2/LT2 (0.5), finally declining even further into an anticorrelated range above VT2/LT2 intensities (below 0.5) (Rogers & Gronwald, 2022). Additionally, DFA a1 is thought to be illustrative of the “Network” theory of exercise, which is a construct blending multiple neuromuscular, biochemical, peripheral, and central nervous system (CNS) inputs, leading to an overall assessment of “organismic demand” (Balagué et al., 2020). Studies to date have generally been consistent with defining the first DFA a1 based heart rate variability threshold (HRVT1) with a value of 0.75 generally coinciding with VT1/LT1 and the second heart rate variability threshold (HRVT2) with a value of 0.5 occurring near the VT2/LT2 (Mateo‐March et al., 2022; Naranjo‐Orellana et al., 2021; Rogers et al., 2021a; Rogers, Giles, et al., 2021; Rogers & Gronwald, 2022; Rogers, Mourot, & Gronwald, 2021; Schaffarczyk et al., 2023). These are dimensionless units that in the case of HRVT1 represent a point midway from well correlated to uncorrelated behavior, and totally uncorrelated (random beat‐to‐beat patterns) in the case of HRVT2.

Previous studies investigating DFA a1 behavior during exercise have used differing cycling ramp protocols with slopes ranging between 7 and 25 W · min^−1^ (Blasco‐Lafarga et al., 2017; Gronwald & Hoos, 2020; Hautala et al., 2003; Mateo‐March et al., 2022; Rogers, Mourot, & Gronwald, 2021; Schaffarczyk et al., 2023). Although gas exchange‐based thresholds have been studied in terms of ramp slope, there is no analogous investigation of ramp protocol effects for any HRV‐based ramp testing. To date, there is no empirical data showing whether the slope of ramp incremental (RI) testing affects HRVT estimation or if there is an optimal RI slope for HRVT derivation. Furthermore, there seems to be no data regarding whether RI slope affects DFA a1 behavior in general. Thus, the aim of the present study was to assess the effects of three different incremental ramp slopes of 15, 30, and 45 W · min^−1^ on the DFA a1‐associated thresholds HRVT1 and HRVT2 as measured by HR and V̇O_2_. We hypothesize that the DFA a1‐related thresholds represented by HR or V̇O_2_ may be affected by steep ramp slopes due to the failure to properly capture rapid HRV change over such short time spans.

## 2 METHODS

### 2.1 Participants

Ten females and 11 males were recruited from the local community. Their age was between 18 and 50, no medical problems were present, none were taking any prescription medications (except oral contraceptive use in some female participants), and they were classed as recreationally trained. Recreationally trained was defined as having a maximal V̇O_2_ of greater than 44.9 mL · kg^−1^ · min^−1^ for males and greater than 36.9 mL · kg^−1^ · min^−1^ for females (De Pauw et al., 2013; Decroix et al., 2016). This study was part of a larger project evaluating the effect of three different ramps (15, 30, and 45 W · min^−1^) on physiological and neuromuscular responses to exercise. This group was also used as the basis for a study evaluating exercise thresholds based on NIRS and DFA a1 data using only the 15 W · min^−1^ data (Fleitas‐Paniagua et al., 2023). The order of RI slope protocol testing was randomized and balanced, with a minimum of 24 h and maximum of 7 days between tests. Some technical issues in the RR signal were experienced in two participants during the 30 and/or 45 W · min^−1^ ramps, and they were excluded from further consideration in the current study leaving a total group of 19. Female participants self‐reported a menstrual cycle length of 28 ± 5 days, and four participants were taking hormonal contraceptives. All tests were performed in an environmentally controlled room (temperature: 18–21°C; humidity 50%–60%). Participants were instructed to avoid any food, caffeinated drinks, or intense physical activity for at least 2, 8, and 24 h before testing, respectively. The PARQ+ 2019 questionnaire was completed before physiologic testing. A written informed consent form was obtained for all participants. All practices were approved by the Conjoint Health Research Ethics Board at the University of Calgary (REB18‐0916).

### 2.2 Data collection

#### 2.2.1 RI testing

Testing was performed on an electromagnetically braked cycle ergometer (Velotron; RacerMate) and consisted of 4 min of baseline cycling at 20 W, 6 min of moderate intensity (60 W for females, 80 W for males), and 4 min at 20 W followed by 15, 30, or 45 W · min^−1^ incremental rate (15w, 30w, 45w) until task failure. The test was stopped when the participant was no longer able to maintain a cycling cadence of at least 60 rpm for more than five successive seconds, or at volitional exhaustion despite verbal encouragement. During the baseline cycling portion, cadence was 60–70 rpm, while during the ramp testing cadence was self‐selected. Participants received visual feedback on their cadence but were blinded to the elapsed time and cycling power.

#### 2.2.2 Gas exchange and ventilatory variables

Gas exchange and ventilatory results were measured breath‐by‐breath using a metabolic cart (Quark; Cosmed). The system was calibrated before all tests according to the manufacture's recommendation and consisted of a low dead space turbine as well as oxygen (O_2_) and carbon dioxide (CO_2_) gas analyzers; a syringe of known volume (3 L) and a gas mixture of known concentration (16% O_2_; 5% CO_2_; balance N_2_), respectively, were utilized for calibration. V̇O_2_ data during the RI test were adjusted by removing data points laying ±3 standard deviation (SD) from the local mean and linearly interpolated to 1 s intervals (Origin; Origin Lab). A 20 s rolling average was used to compute the V̇O_2_ values with the highest value of the 20 s values considered as V̇O_2MAX_. The GET determined to occur at the point at which: (i) carbon dioxide production (V̇CO_2_) began to increase disproportionally in relation to V̇O_2_, (ii) a systematic rise in the ventilation (V̇E) versus V̇O_2_ relationship and partial pressure of expired oxygen (P_E_O_2_) occurred, and (iii) there was stability in the ventilatory equivalent of V̇CO_2_ (V̇E/V̇CO_2_) and partial pressure of expired carbon dioxide (P_E_CO_2_) (Beaver et al., 1986; Keir et al., 2022; Poole & Jones, 2012). The RCP corresponded to the second disproportional increase (second breakpoint) in the V̇E/V̇O_2_ relationship, where the P_E_CO_2_ began to fall after a period of isocapnic buffering (Keir et al., 2022; Poole & Jones, 2012). The relationship between V̇E/V̇CO_2_ against V̇O_2_ was also used for verification of the RCP. The average value from three evaluators was used for the GET and RCP. If the evaluators had a disagreement of more than 100 mL ∙ min^−1^ in the result, a second round of evaluation was performed together until a consensus was reached.

#### 2.2.3 RR measurements and HRVT estimation

The Polar H10 chest strap (Polar Electro) with a sampling rate of 1000 Hz was used to record the RR time series of each participant. The strap electrodes were covered with conductive gel and securely fitted to the sub‐pectoral area with the module initially centered over the sternum. Prior to data recording, the Polar H10 ECG waveform was visually evaluated with an Android app based upon the Polar API, ECG Logger (https://ecglogger.en.aptoide.com/app). The chest strap was shifted slightly to the left if the R peak amplitude was lower than the S wave in order to optimize DFA a1 measurements. Output was transmitted via Bluetooth to an Android smartphone running an open source recording application (FatMaxxer, https://github.com/IanPeake/FatMaxxer) and stored as .csv files for further analysis. Data was further processed by Kubios HRV Software (Version 3.5, Biosignal Analysis and Medical Imaging Group, Department of Physics, University of Kuopio, Kuopio, Finland). Kubios preprocessing settings were kept at the default values including the RR detrending method which was set at “Smoothness priors” (Lambda = 500). DFA a1 window width was changed from its default to 4 ≤ *n* ≤ 16 beats (Rogers & Gronwald, 2022). Visual inspection of the entire test recording was done to determine missing beat artifact, sample quality, noise, and any arrhythmia. The RR series was corrected by the Kubios “automatic method” (Lipponen & Tarvainen, 2019) and applicable results exported for further analysis. Acceptable percent artifact during threshold interpretation segments was set to below 5% (Rogers et al., 2021b). Two participants with excessive atrial, ventricular ectopy, and/or artifact above 5% were excluded from analysis, leaving a total of 17 (9 male, 8 female). Maximal HR was calculated as the highest value from a 20 s rolling average.

**FIGURE 1:**
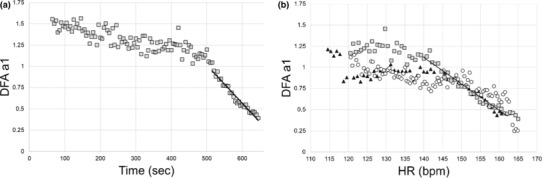
Plotting of DFA a1 over time in seconds during a 30 W · min^−1^ RI (a) and DFA a1 versus HR (bpm) in a representative participant performing three types of incremental cycling ramp tests (b). Circle: 15 W · min^−1^; Square: 30 W · min^−1^; Triangle: 45 W · min^−1^ RI slopes. The solid line denotes the line of regression for heart rate variability threshold assessment for the 30 W·min^−1^ ramp values. DFA, detrended fluctuation analysis; HR, heart rate; RI, ramp incremental.

The following process (Rogers et al., 2021a; Rogers, Giles, et al., 2021) was used to indicate at what level of cycling intensity (as V̇O_2_ or HR) the DFA a1 would cross a value of 0.75 to define the HRVT1 and 0.5 for the HRVT2: DFA a1 was calculated from the RI test RR series using 2 min time windows with a recalculation every 5 s throughout the test. This method of repeat, rolling recalculation is known as the “time varying” option available in Kubios HRV software. Two‐minute time windowing was chosen based on the beat count required for valid results (Chen et al., 2002; Hautala et al., 2003; Shaffer et al., 2020). Each DFA a1 value is based on the RR series 1 min pre and 1 min post the specified time stamp. For example, at a time stamp 6 min into the testing, the DFA a1 is calculated from the 2 min window starting from Minute 5 and ending at Minute 7 and labeled as the DFA a1 at 6 min elapsed. Plotting of DFA a1 versus time was then performed (Figure 1a). Inspection of the DFA a1 relationship with time generally showed a reverse sigmoidal curve with a stable area above 1.0 at low work rates, a rapid, near linear drop reaching below 0.5 at higher intensity, then flattening without major change. A linear regression was done on the subset of data consisting of the rapid decline from values near 1.0 (correlated) to approximately 0.5 (uncorrelated) or below. The time of DFA a1 reaching 0.75 or 0.5 was calculated based on the equation from that linear section. The time of DFA a1 reaching 0.75 or 0.5 was then converted to V̇O_2_ using the V̇O_2_ versus time relation from the corresponding gas exchange test, resulting in the V̇O_2_ at which DFA a1 equaled 0.75 (HRVT1) or 0.5 (HRVT2). A different method was used to determine the HR reached at a DFA a1 of 0.75 or 0.5. DFA a1 and HR data from each 2 min rolling window was used to plot the average HR versus DFA a1 over the same elapsed frame as used in the V̇O_2_ calculation. The HR at which DFA a1 equaled 0.75 or 0.5 was found using the same technique as above, a linear regression through the rapid change section of DFA a1 values of 1.0 to below 0.5, with a subsequent equation for HR and DFA a1 (Figure 1b).

### 2.3 Statistical analysis

Normality of data was assessed by Shapiro–Wilk's testing and inspection of histograms. Data were reported as means ± SD. The correlation between a given ramp's HRVT1 and HRVT2 (HR and the V̇O_2_ responses) with another (e.g., 15 vs. 30 W · min^−1^) were assessed using Pearson's *r* coefficient and standard error of estimate (SEE). The agreement was evaluated with Bland–Altman analysis (Bland & Altman, 1999) with limits of agreement (LoA) (±2 SD). Examination of the distribution of the mean differences in the Bland–Altman analysis was made to confirm normality and if proportional bias was detected, a regression‐based calculation of mean differences and LoA were presented (Ludbrook, 2010). Pearson's *r* strength of correlation was evaluated as follows: 0.3 ≤ *r* < 0.5 low; 0.6 ≤ *r* < 0.8 moderate and *r* ≥ 0.8 high (Chan, 2003). Comparisons between select variables were made using paired *t*‐test with a *p* ≤ 0.05 as statistically significant. Intraclass correlation coefficient (ICC_3,1_) with 95% confidence intervals (CIs) was calculated across the three‐ramp series of HRVT1, HRVT2 for both HR, V̇O_2_. ICC_3,1_ correlation strength was classified as according to the following, <0.40 as poor, 0.40 to 0.59 as fair, 0.60 to 0.74 as good, and 0.75 to 1.00 as excellent (Cicchetti, 1994). Single factor, repeated‐measures ANOVA was performed across the three ramp series of HRVT1, HRVT2 for both HR, V̇O_2_. Analysis was performed using Microsoft Excel 365 with Real Statistics Resource Pack software (Release 6.8) and Analyse‐it software (Version 6.01).

## 3 RESULTS

### 3.1 Baseline participant demographic and physiologic data

A summary of male, female and group physical characteristics are presented in Table 1 along with the HR_MAX_, V̇O_2MAX_, GET/RCP, HRVT1/2 V̇O_2,_ and HR from the 15 W · min^−1^ ramp test, with partial results from participants in this study previously reported (De Pauw et al., 2013).

**TABLE 1:** Participants characteristics: age (years), weight, (kg), HR_MAX_ (bpm), V̇O_2MAX_ (mL · kg^−1^ · min^−1^), GET V̇O_2_ (mL · kg^−1^ · min^−1^), GET HR (bpm), RCP V̇O_2_ (mL · kg^−1^ · min^−1^), RCP HR (bpm), HRVT1 V̇O_2_ (mL · kg^−1^ · min^−1^), HRVT1 HR (bpm), HRVT2 V̇O_2_ (mL · kg^−1^ · min^−1^), HRVT2 HR (bpm) derived from the 15 W · min^−1^ ramp as mean ± standard deviation (SD).

	Male (*N* = 9)	Female (*N* = 8)	Group (*N* = 17)
Age (years)	35 ± 9	33 ± 10	34 ± 9
Weight (kg)	76 ± 13	63 ± 5	70 ± 12
HR_MAX_ (bpm)	176 ± 15	183 ± 7	179 ± 12
V̇O_2MAX_ (mL · kg^−1^ · min^−1^)	53.8 ± 10.1	41.4 ± 9.0	48.0 ± 11.4
GET V̇O_2_ (mL · kg^−1^ · min^−1^)	31.9 ± 8.8	26.4 ± 4.0	29.3 ± 7.5
GET HR (bpm)	124 ± 17	137 ± 12	130 ± 16
RCP V̇O_2_ (mL · kg^−1^ · min^−1^)	46.2 ± 9.0	35.4 ± 7.7	41.2 ± 10.0
RCP HR (bpm)	156 ± 16	162 ± 11	159 ± 14
HRVT1 V̇O_2_ (mL · kg^−1^ · min^−1^)	38.5 ± 6.7	31.9 ± 7.3	35.4 ± 7.7
HRVT1 HR (bpm)	146 ± 14	154 ± 9	150 ± 13
HRVT2 V̇O_2_ (mL · kg^−1^ · min^−1^)	45.0 ± 6.8	35.9 ± 7.9	40.7 ± 8.6
HRVT2 HR (bpm)	159 ± 16	164 ± 8	161 ± 13

Abbreviations: GET, gas exchange threshold; HR, heart rate; HRV, heart rate variability; HRVT, heart rate variability threshold; RCP, respiratory compensation point.

### 3.2 HRVT1, HRVT2 across the three ramps

**FIGURE 2:**
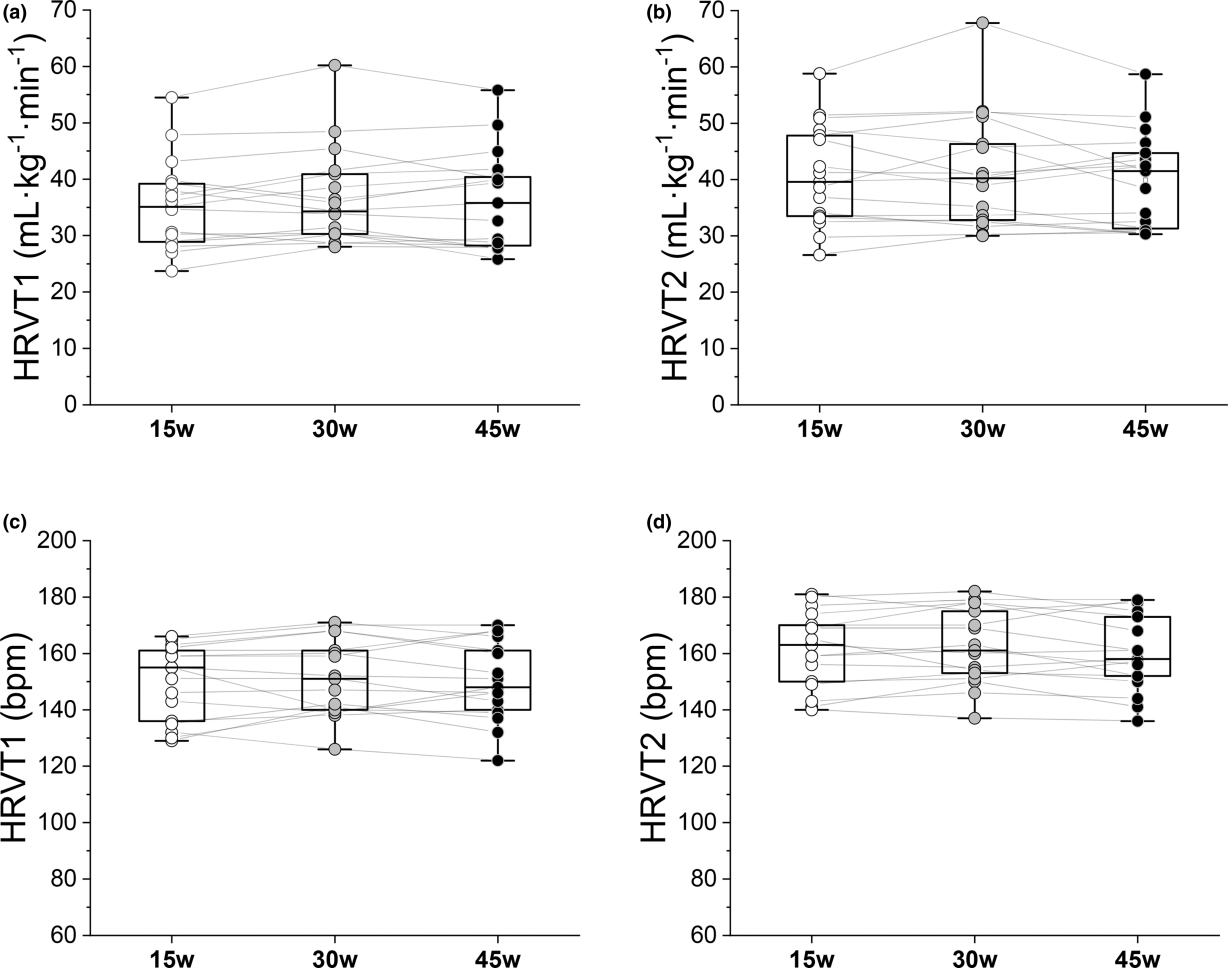
Box and whisker plots of individual participant ramp responses: HRVT1 (a, c); HRVT2 (b, d); both as either V̇O_2_ (mL · kg^−1^ · min^−1^) or HR (bpm); box edges represent first and third quartiles, the central line is the median, and the whiskers are the minimum and maximum values; 15 W · min^−1^ (15w), 30 W · min^−1^ (30w), and 45 W · min^−1^ (45w) refer to ramp slope. *N* = 17. HRVT, heart rate variability threshold.

**FIGURE 3:**
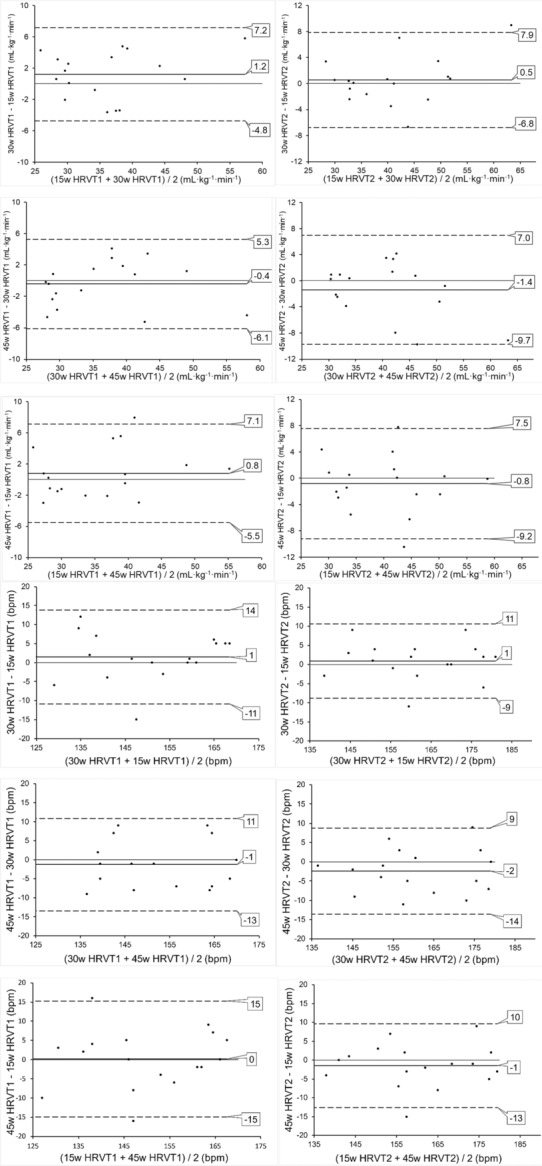
Bland–Altman plots of ramp responses: HRVT1; HRVT2; both as either V̇O_2_ (mL · kg^−1^ · min^−1^) or heart rate (bpm); mean; SD (standard deviation); 15 W · min^−1^ (15w), 30 W · min^−1^ (30w), and 45 W · min^−1^ (45w) refer to ramp slope. Mean bias (solid line) and limits of agreement (dashed line) indicated. *N* = 17. HRVT, heart rate variability threshold.

Participants HRVT1, HRVT2 (as V̇O_2_ and HR) for each ramp with group mean and SD are shown in Figure 2 with individual detailed data in Table S1. Paired *t*‐testing between each slope group (e.g., 15w vs. 30w, 30w vs. 45w, or 15w vs. 45w) showed no significant differences in the mean V̇O_2_ or HR in either HRVT1 or HRVT2 (*p* > 0.1 or higher). Correlation values between ramps using Pearson's correlation coefficient are shown in Table 2 along with SEE with detailed regression plots in Figure S1. A summary of Bland–Altman analysis is also shown in Table 2 with mean bias and SD (LoA = SD × 2). Detailed Bland–Altman plots for V̇O_2_ and HR responses (mean bias with limits of agreement) are shown in Figure 3. There was no evidence of proportional bias (change in the bias), heteroscedasticity (change in scatter of differences) over the V̇O_2,_ or HR ranges. An excellent degree of reliability was seen across all three ramps, with an ICC_3,1_ of 0.93 (CI: 0.86–0.97, *p* = 0.05) and 0.88 (CI: 0.75–0.95, *p* = 0.05) for the HRVT1 V̇O_2_ and HRVT1 HR respectively. This close relationship continued to the HRVT2 with ICC_3,1_ measurements of 0.90 (CI: 0.80–0.96, *p* = 0.05) and 0.92 (CI: 0.83–0.97, *p* = 0.05) for the HRVT2 V̇O_2_ and HRVT2 HR, respectively. Single factor repeated‐measures ANOVA did not show any statistical differences across the three RI groups, with *F* = 1.4, *p* = 0.27 and *F* = 0.5, *p* = 0.62 for HRVT1 V̇O_2_ and HRVT1 HR respectively and *F* = 1.0, *p* = 0.39 and *F* = 1.7, *p* = 0.20 for HRVT2 V̇O_2_ and HRVT2 HR respectively.

**TABLE 2:** Pearson's *r*; standard error of estimate (SEE); mean bias with standard deviation (SD) as either V̇O_2_ or HR for ramp comparisons for all participants.

	15w vs. 30w	30w vs. 45w	15w vs. 45w
Correlation			
HRVT1 V̇O_2_ *r* (SEE)	0.94 (3.1)	0.95 (3.0)	0.93 (3.3)
HRVT1 HR *r* (SEE)	0.89 (6)	0.90 (6)	0.84 (8)
HRVT2 V̇O_2_ *r* (SEE)	0.93 (3.8)	0.91 (3.6)	0.88 (4.2)
HRVT2 HR *r* (SEE)	0.93 (5)	0.91 (6)	0.91 (6)
Bland–Altman			
HRVT1 V̇O_2_ bias (SD) (mL · kg^−1^ · min^−1^)	1.2 (3.0)	−0.4 (2.9)	0.8 (3.2)
HRVT1 HR bias (SD) (bpm)	2 (6)	−1 (6)	0 (8)
HRVT2 V̇O_2_ bias (SD) (mL · kg^−1^ · min^−1^)	0.5 (3.7)	−1.4 (4.2)	−0.8 (4.3)
HRVT2 HR bias (SD) (bpm)	1 (5)	−2 (6)	−1 (6)

*Note*: 15 W · min^−1^ (15w), 30 W · min^−1^ (30w), and 45 W · min^−1^ (45w) refer to ramp slope.

Abbreviations: HR, heart rate; HRVT, heart rate variability threshold.

## 4 DISCUSSION

Over the past 20 years numerous studies evaluating DFA a1 behavior during dynamic exercise have been performed (Blasco‐Lafarga et al., 2017; Gronwald et al., 2020; Gronwald & Hoos, 2020; Hautala et al., 2003; Mateo‐March et al., 2022; Naranjo‐Orellana et al., 2021; Rogers et al., 2021a; Rogers, Giles, et al., 2021; Rogers & Gronwald, 2022; Rogers, Mourot, & Gronwald, 2021; Schaffarczyk et al., 2023). However, despite showing potential as a marker defining exercise thresholds through RI testing, there has been no consensus as to what type of ramp protocol is optimal or desirable. Therefore, the intent of this study was to assess the behavior of DFA a1‐related HRVTs during cycling RI with varying slopes. Literature has shown that fast ramps tend to have the greatest degree of discordance between measurements such as cycling power and corresponding gas exchange‐derived thresholds (Boone & Bourgois, 2012; Iannetta et al., 2019; Keir et al., 2018; Weston et al., 2002), unless a correction is used to account for the V̇O_2_ mean response time and slow component (Iannetta et al., 2020; Keir et al., 2018). In the context of an established ANS marker such as DFA a1, conjecture as to the effect of ramp slope is complex. A slower incremental rise in work rate resulting in a longer ramp may lead to fatigue‐related effects (Rogers, Mourot, Doucende, et al., 2021; Schaffarczyk et al., 2022) that could result in biased threshold estimation. On the other hand, a rapid intensity rise may not be able to truly describe an index encompassing a measuring window of 2 min. For example, over the 2‐min DFA a1 measuring window, a full 90 W of external load increase will have occurred if the RI test was performed at a 45 W · min^−1^ slope. Whether or not DFA a1 values done under such non‐steady‐state circumstances produce comparable results to those done under a more gradual rise in load is unclear.

Since DFA a1 calculations need about a 2‐min measurement window for validity (Chen et al., 2002; Hautala et al., 2003; Shaffer et al., 2020), fast ramps lasting only several minutes may also present a challenge simply on the basis of limited available data points. In addition, even though the ANS response is believed to be rapid in relation to the various regulation factors (Devarajan et al., 2022; Ernst, 2017; Gourine et al., 2016), there could be a lag between these inputs and their effect on DFA a1 behavior during fast ramps. Many initial studies measured DFA a1 toward the end of a “step” interval of varying length but always longer than 2‐min steps (Blasco‐Lafarga et al., 2017; Gronwald & Hoos, 2020; Hautala et al., 2003). When DFA a1 was first proposed as a surrogate marker for ventilatory threshold determination (Rogers, Giles, et al., 2021), a new calculation technique was used, based on the “time varying” method available in Kubios HRV software. Time varying refers to the index being recalculated continually every 5 s throughout the exercise period. Before this technique, the index was determined either at the end of each interval step or at periodic, non‐overlapping points during the exercise test. Since we are now able to easily measure DFA a1 on a more granular level over the course of increasing load, the question remains whether absolute ramp slope matters for both index behavior and HRVT determination.

The results of this study show that the V̇O_2_ or HR reached at both HRVT1 and HRVT2 is relatively independent of the ramp slope during incremental exercise testing (for those slopes used in this report). There was excellent correlation between all three ramp protocols using ICC_3,1_ with values between 0.88 and 0.93 and no mean differences across all groups with ANOVA. Pearson's *r* was also highly correlated between paired ramp groups with values between 0.84 and 0.95 (Table 2). Bland–Altman analysis showed small mean differences between ramp slopes (Table 2; Figure 3). There were no statistical differences seen between any ramp slope series looking at either HR or V̇O_2_ according to paired *t*‐testing. Importantly, there was no major discrepancy in correlation/agreement or *t*‐testing in comparing the 15 to the 45 W · min^−1^ ramp slopes, despite the three‐fold difference in power output rate increment. The observation that DFA a1 is capable of rapidly shifting during the 45 W · min^−1^ ramp to match that of the 15 W · min^−1^ ramp is a novel finding of interest. Like the HR response to RI testing (Davis et al., 1982; Weston et al., 2002), there appears to be a prompt matching of “organismic” demand as represented by DFA a1, to the external exercise load. This makes sense as both HR and HRV responses are mediated by related and/or linked ANS, CNS centers, vagal output, and effects on the atrial pacemaker cells (Devarajan et al., 2022; Ernst, 2017; Gourine et al., 2016; Michael et al., 2017; White & Raven, 2014). However, it has been unclear whether an HRV measurement window encompassing a relatively large span of differing metabolic input would yield usable results. This similarity in DFA a1 response across disparate ramp slopes is illustrated in a detailed plot of HR versus DFA a1 of a typical participant during the three RI tests (Figure 1b). The pattern of DFA a1 decline as HR rises is similar across the differing ramp slopes. Since the 45 W · min^−1^ group had similar agreement to that of the 15 or 30 W · min^−1^ groups, it seems that DFA a1 measurement of a linear increasing load leads to comparable HR or V̇O_2_ correspondence no matter the rate of rise (within tested limits). This has major practical significance as prior and possibly future studies evaluating DFA a1 behavior may employ RI with different slopes. Since it appears the RI slope does not affect the resultant HRVTs, these studies can be more easily compared and implemented.

### 4.1 Limitations and future directions

As previously reviewed (Rogers & Gronwald, 2022), artifact correction bias, arrhythmia, device bias, and electrocardiogram (ECG) waveform can affect both absolute DFA a1 values and HRVT levels. In this study, attempts were made to optimize ECG waveform amplitude and acceptable artifact correction was below 5%. Additionally, the presence of fatigue, stress, and hormonal influence can theoretically contribute to HRVT variation due to effects on the ANS (Rogers, Mourot, Doucende, et al., 2021; Schaffarczyk et al., 2022; Stanley et al., 2013). Therefore, it is possible some test‐to‐test disparity was caused by differences in daily stress levels. With respect to HRVT concordance to ventilatory/lactate threshold parameters, there was a much higher bias seen in this study with respect to HRVT1 than HRVT2 as noted in Table 1 (Mateo‐March et al., 2022; Naranjo‐Orellana et al., 2021; Rogers, Giles, et al., 2021; Rogers, Mourot, et al., 2021; Schaffarczyk et al., 2023). This may have been due to five participants having GET‐related HR below 120 bpm including one at 93 bpm. The underlying reason for the GET:HRVT1 discordance is unclear, but further evaluation of HRVT1 in populations with relatively low GET‐related HR could be helpful. As reported elsewhere (Fleitas‐Paniagua et al., 2023; Mateo‐March et al., 2022; Naranjo‐Orellana et al., 2021; Rogers et al., 2021b; Schaffarczyk et al., 2023), excellent agreement with the RCP/VT2 and HRVT2 was seen. Lastly, similar RI comparison studies in more focused populations such as the very young, elderly, and elite athletes could be helpful as well.

### 4.2 Perspectives and significance

The current results indicate that the HRV threshold based on the nonlinear index DFA a1, behaves in a comparable fashion across incremental cycling ramps protocols of 15, 30, and 45 W · min^−1^. There was no apparent difference in HRVT1 or HRVT2 response as measured by HR and V̇O_2_ comparisons. Despite a three‐fold difference in work rate increment, HRVT response was equivalent, indicating that there is a rapid matching of “organismic” demand as represented by DFA a1, to the external exercise load. Given this result, both past and future ramp studies examining HRVTs can now be reliably performed and compared without major concern for the incremental slope employed.

## AUTHOR CONTRIBUTIONS

Bruce Rogers, Pablo R. Fleitas‐Paniagua, and Juan M. Murias conceived the study. Pablo R. Fleitas‐Paniagua, Rafael de Almeida Azevedo, and Mackenzie Trpcic performed the data collection. Bruce Rogers wrote the first draft of the article. Pablo R. Fleitas‐Paniagua, Rafael de Almeida Azevedo, Juan M. Murias, and Bruce Rogers performed the data analysis. All authors revised and approved the final version.

## FUNDING INFORMATION

Dr. Juan M. Murias' work was supported by the Natural Sciences and Engineering Research Council of Canada (RGPIN‐2016‐03698) and the Heart & Stroke Foundation of Canada (1047725). Pablo R. Fleitas‐Paniagua was supported by Consejo Nacional de Ciencia y Tecnología, Paraguay.

## CONFLICT OF INTEREST STATEMENT

The authors declare that there was no conflict of interest, financial, or otherwise at the moment of submission of this manuscript.

## Supporting information


Data S1.

## Data Availability

The datasets analyzed during the current study are available from the corresponding author on reasonable request.
