# Supplementary material for: Effect of ramp slope on intensity thresholds based on correlation properties of heart rate variability during cycling
Source: Physiol Rep. 2023 Aug 7;11(15):e15782. doi: 10.14814/phy2.15782 (PMC10406567; doi:10.14814/phy2.15782)

Supplemental Table 1 and Figure 1

Table 1 – Individual Participant Ramp Responses: M (male); F (female); HRVT1 (heart rate variability threshold 1); HRVT2 (heart rate variability threshold 2); both as either V̇O_2_ (mL·kg^-1^·min^-1^) or HR (bpm); mean; SD (standard deviation); 15 W·min^-1^ (15w), 30 W·min^-1^ (30w), 45 W·min^-1^ (45w) refer to ramp slope.

|  | Sex (M/F) | HRVT1 V̇O2 (mL·kg^-1^·min^-1^) | | | HRVT2 V̇O2 (mL·kg^-1^·min^-1^) | | | HRVT1 HR (bpm) | | | HRVT2 HR (bpm) | | |
| --- | --- | --- | --- | --- | --- | --- | --- | --- | --- | --- | --- | --- | --- |
|  |  | 15w | 30w | 45w | 15w | 30w | 45w | 15w | 30w | 45w | 15w | 30w | 45w |
|  | M | 28.8 | 30.4 | 25.8 | 36.8 | 35.1 | 31.3 | 161 | 161 | 168 | 181 | 175 | 178 |
|  | M | 54.5 | 60.2 | 55.8 | 58.8 | 67.8 | 58.7 | 129 | 141 | 132 | 141 | 150 | 141 |
|  | M | 35.1 | 38.5 | 40.4 | 39.6 | 40.2 | 43.6 | 165 | 170 | 170 | 177 | 179 | 179 |
|  | M | 37.9 | 34.3 | 35.8 | 41.2 | 41.1 | 42.5 | 132 | 126 | 122 | 140 | 137 | 136 |
|  | M | 34.6 | 33.8 | 32.6 | 48.8 | 46.3 | 38.4 | 163 | 168 | 161 | 180 | 182 | 175 |
|  | M | 36.1 | 40.9 | 41.7 | 51.4 | 52.1 | 48.9 | 130 | 139 | 146 | 156 | 155 | 158 |
|  | M | 43.1 | 45.4 | 40.1 | 47.8 | 51.2 | 41.5 | 136 | 138 | 140 | 143 | 146 | 144 |
|  | M | 39.7 | 36.4 | 39.3 | 42.3 | 38.9 | 42.4 | 155 | 152 | 151 | 163 | 160 | 161 |
|  | M | 37.0 | 41.5 | 44.9 | 38.6 | 45.7 | 46.5 | 143 | 139 | 148 | 150 | 151 | 157 |
|  | F | 30.6 | 28.6 | 29.4 | 34.0 | 31.6 | 32.5 | 155 | 140 | 139 | 165 | 154 | 150 |
|  | F | 28.9 | 31.4 | 27.7 | 33.5 | 33.6 | 34.0 | 135 | 142 | 137 | 149 | 153 | 152 |
|  | F | 47.8 | 48.4 | 49.6 | 50.9 | 51.9 | 51.1 | 151 | 151 | 143 | 159 | 163 | 152 |
|  | F | 27.0 | 30.1 | 27.8 | 32.4 | 32.8 | 30.4 | 159 | 160 | 153 | 169 | 169 | 161 |
|  | F | 28.0 | 28.6 | 28.2 | 29.7 | 30.2 | 30.5 | 146 | 147 | 146 | 159 | 161 | 156 |
|  | F | 39.2 | 35.8 | 39.9 | 47.1 | 40.5 | 44.7 | 166 | 171 | 166 | 174 | 178 | 173 |
|  | F | 23.7 | 28.0 | 27.8 | 26.6 | 30.0 | 30.9 | 159 | 159 | 168 | 170 | 170 | 179 |
|  | F | 30.2 | 30.3 | 28.7 | 33.2 | 32.4 | 30.3 | 162 | 168 | 160 | 169 | 178 | 168 |
| Mean ±(SD) | | 35.4 ± 7.7 | 36.6 ± 8.3 | 36.2 ± 8.5 | 40.7 ± 8.6 | 41.3 ± 9.9 | 39.9 ± 8.3 | 150 ± 13 | 151 ± 13 | 150 ± 14 | 161 ± 13 | 162 ± 13 | 160 ± 13 |

Figure 1 – Regression analysis of individual HRVT1 and HRVT2 participant responses for 15 W·min^-1^ (15w), 30 W·min^-1^ (30w), 45 W·min^-1^ (45w) ramp slopes as either V̇O_2_ (mL·kg^-1^·min^-1^) or HR (bpm).


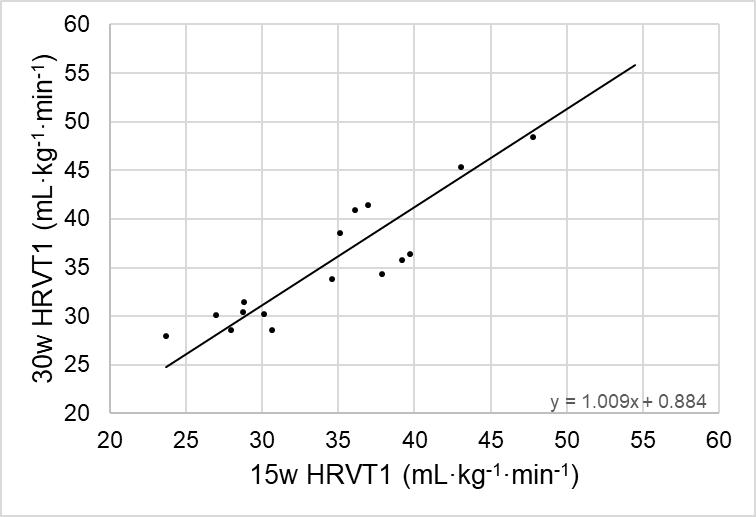

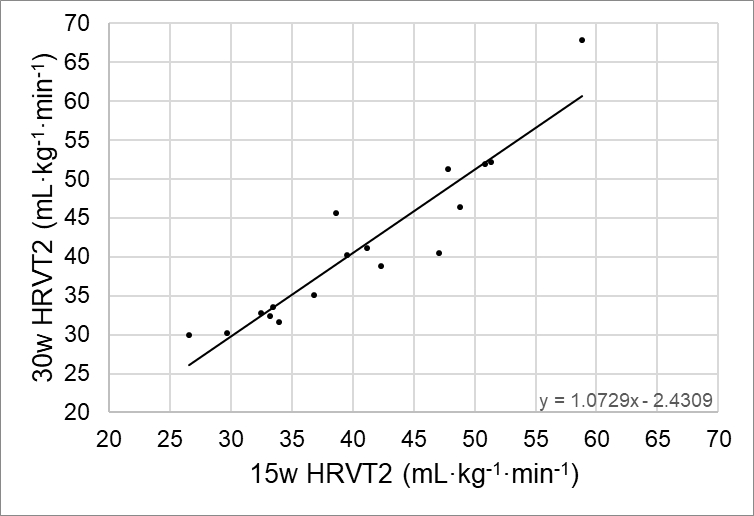


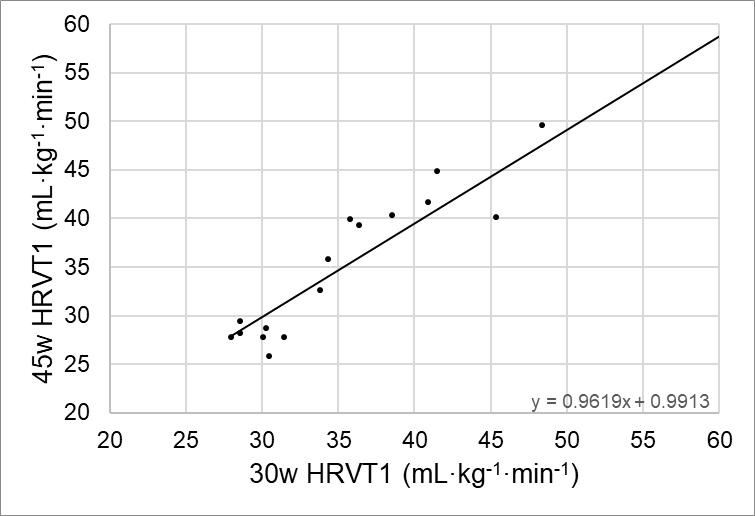

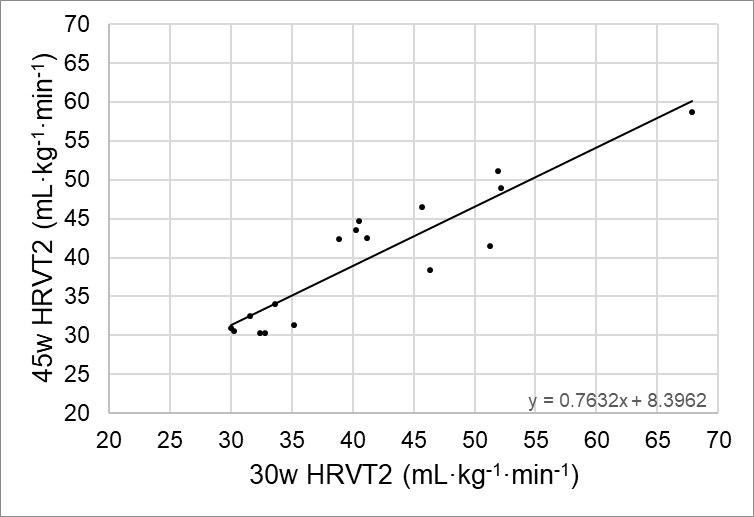


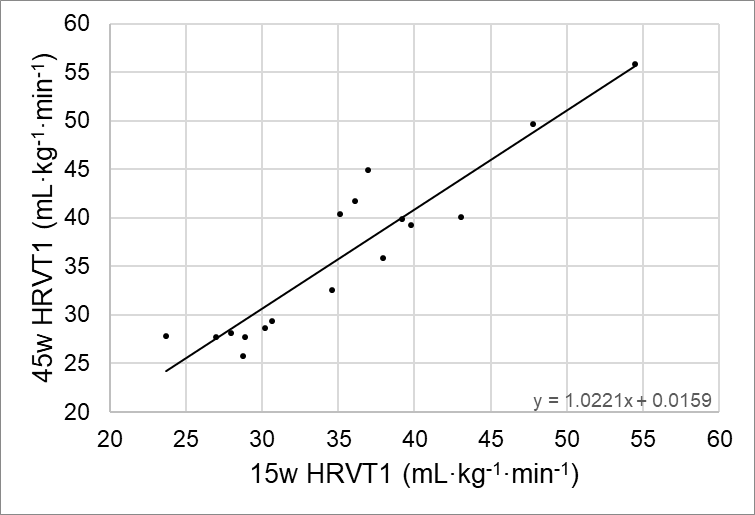

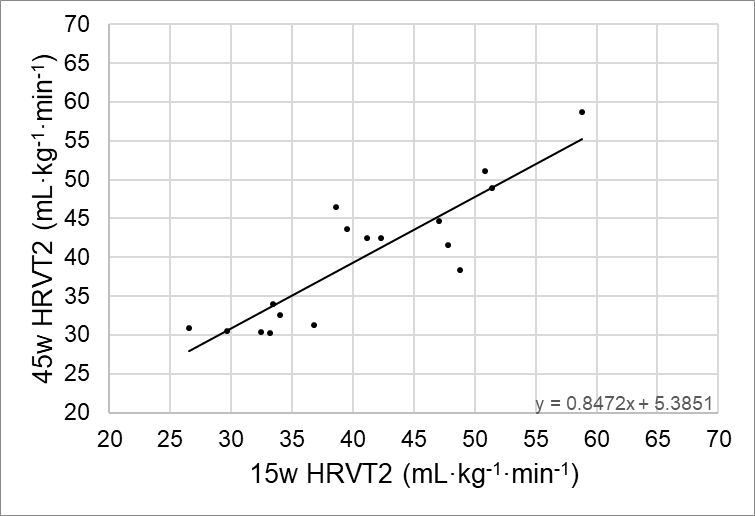


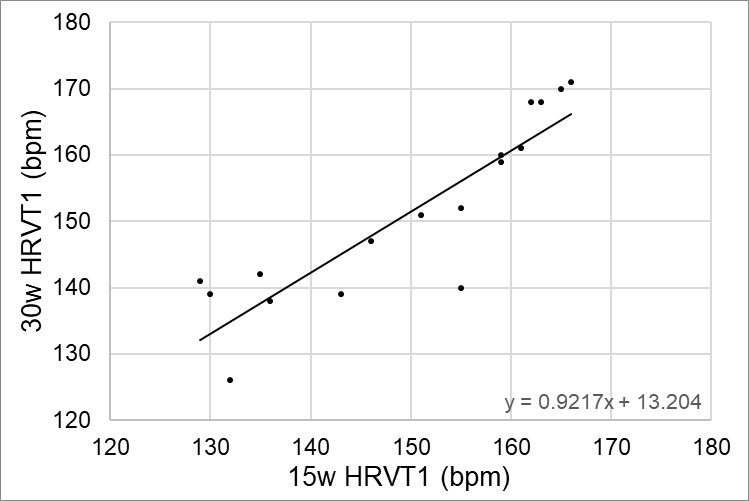

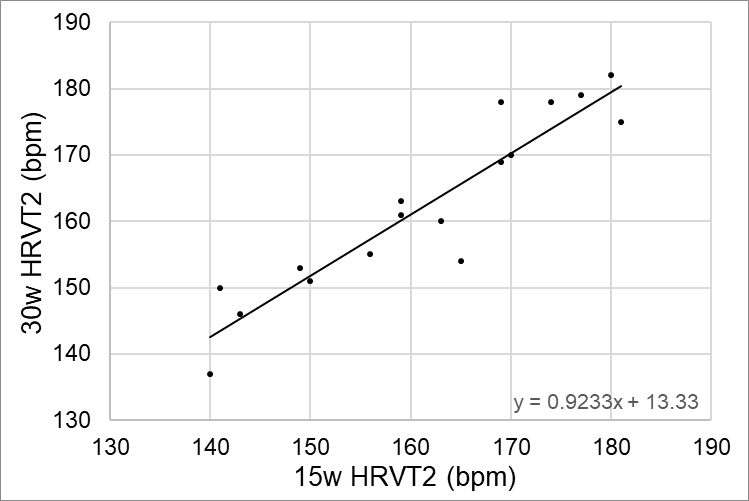


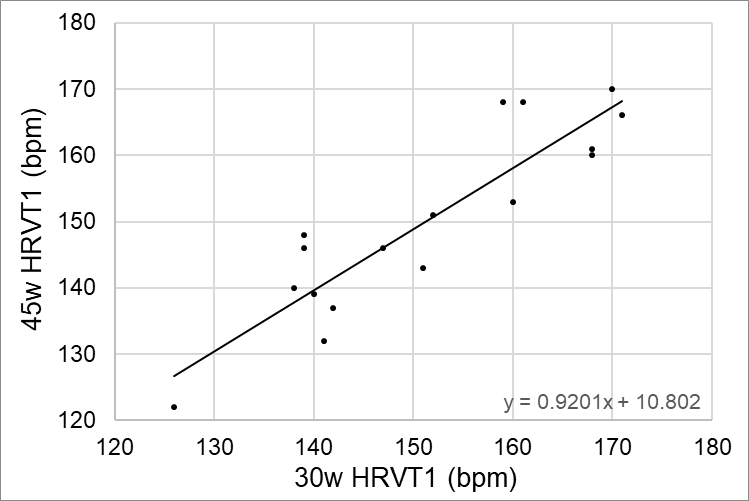

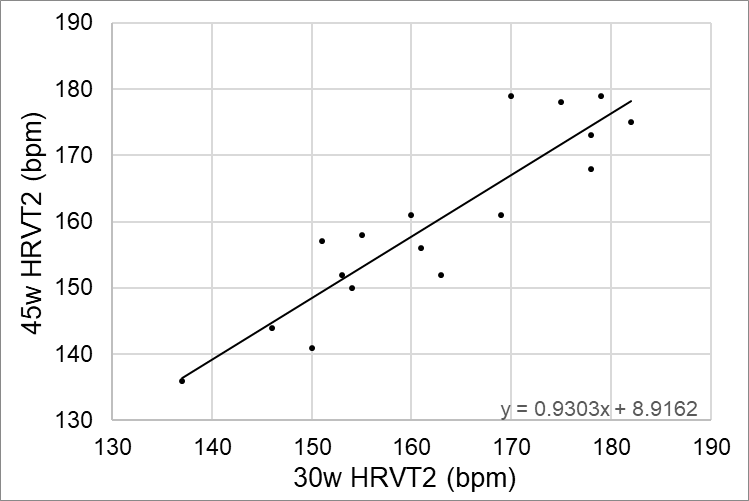


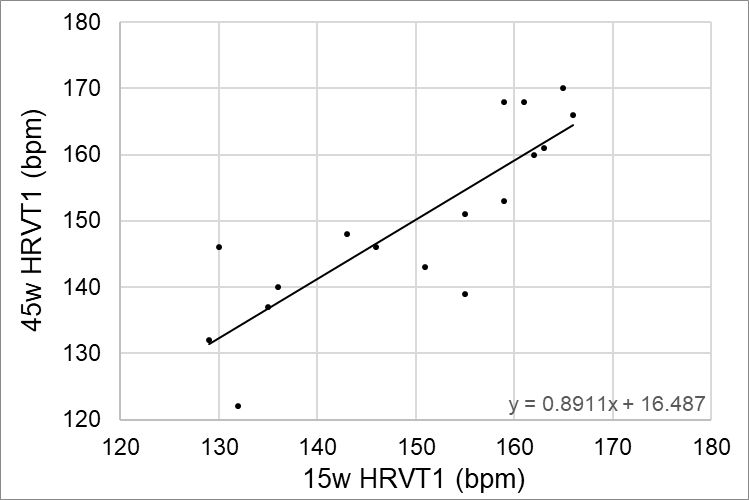

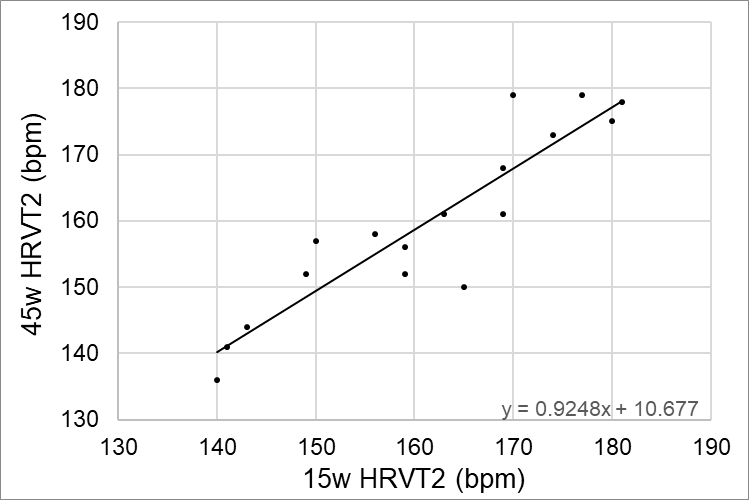

Supplement: Supplementary file 1 — Data S1. [file PHY2-11-e15782-s001.docx]
